# From Mr. Herbert J. Paterson, to the Editor, "The Times."

**Published:** 1919-06-07

**Authors:** Herbert J. Paterson

**Affiliations:** Medical Hon. Secretary, Royal British Nurses' Association, and Hon. Treasurer, Central Committee for State Registration of Trained Nurses.


### NURSES' REGISTRATION.

### The Central Committee's Bill.

### TWO LETTERS OF SPECIAL INTEREST.

### From Mr. Herbert J. Paterson, to the Editor, "The Times."

Sir,—Will you allow me the courtesy of your columns to correct an error in the letter from the Hon. Sir Arthur Stanley which appeared in your issue of May 26?

Sir Arthur Stanley states that the actual number of nurses belonging to the group of societies affiliated to the Central Committee for the State Registration of Trained Nurses is certainly under half the number of those already on the College Register. This statement is quite erroneous, as the Central Committee represents at least 13,000 trained nurses.

Sir Arthur, moreover, omits to mention that the Central Committee includes delegates from the British Medical Association representing over 20,000 medical men.

During the debate in the House of Lords and in the Standing Committee of the House of Commons, attention was called to the methods adopted to induce nurses to enrol themselves on the Register of the College of Nursing. The question of numbers is, however, a side issue. The promoters of the Central Committee's Bill base their case not merely on numerical strength, but on the principles of right and justice, and we maintain that, following the precedent of the General Medical Council, there should be a similar statutory body for the nursing profession, whose powers should be limited to the keeping of a State Register, the supervision of examinations, and defining the necessary curriculum. This will be quite enough for one body to accomplish.

The Bill promoted by the College of Nursing aims at combining the functions of the Royal College of Surgeons, the General Medical Council, a university, and a benevolent corporation all in one body. Nursing is a profession, and as such should have self-government by professional persons. The authorities of the nurses' training-schools should have no place on the body which defines the standards and curricula required. The managers of hospitals might just as well claim to be represented on the General Medical Council.

In a word, the College Bill is the employers' Bill; the Central Committee's Bill is a measure to secure to the nursing profession that self-government and economic independence which is their just right and due.

I am, yours faithfully,

Herbert J. Paterson, Medical Hon. Secretary, Royal British Nurses' Association, and Hon. Treasurer, Central Committee for State Registration of Trained Nurses.

(*The Times*, June 2.)

